# Effective Removal of Cd from Aqueous Solutions Using P-Loaded Ca-Mn-Impregnated Biochar

**DOI:** 10.3390/molecules28227553

**Published:** 2023-11-12

**Authors:** Cheng Qiu, Chengwei Wang, Qinghai Liu, Minling Gao, Zhengguo Song

**Affiliations:** 1 Institute of Agricultural Product Quality Standard and Testing Research, Tibet Academy of Agricultural and Animal Husbandry Sciences, Lhasa 850032, China; chengqiu_2006@163.com (C.Q.); song199611@tom.com (Q.L.); 2 Department of Civil and Environmental Engineering, Shantou University, No. 243 Daxue Road, Shantou 515063, China; wangcw96@163.com; 3 School of Environmental Science and Engineering, Tiangong University, No. 399 Binshui West Road, Xiqing District, Tianjin 300387, China; 4 Guangdong Provincial Key Laboratory of Marine Disaster Prediction and Prevention, Shantou University, Shantou 515063, China

**Keywords:** phosphate-loaded Ca-Mn-impregnated biochar, adsorption, cadmium, mechanism, aqueous solutions

## Abstract

Cadmium (Cd) pollution in wastewater has become an increasingly widespread concern worldwide. Studies on Cd (II) removal using phosphate-adsorbed sorbents are limited. This study aimed to elucidate the behaviors and mechanisms of Cd (II) sorption on phosphate-loaded Ca-Mn-impregnated biochar (P_s_-CMBC). The Cd (II) sorption on P_s_-CMBC reached equilibrium within 2 h and exhibited a higher sorption efficiency than biochar and CMBC. Additionally, the Langmuir isotherm could better describe the Cd (II) adsorption on the sorbents. P_75_-CMBC had a maximum Cd (II) sorption capability of 70.13 mg·g^−1^ when fitted by the Langmuir isotherm model, which was approximately 3.18 and 2.86 times greater than those of biochar and CMBC, respectively. Higher pH (5–7) had minimal effect on Cd (II) sorption capacity. The results of characterization analyses, such as SEM-EDS, FTIR, and XPS, suggested that there was a considerable difference in the sorption mechanisms of Cd (II) among the sorbents. The primary sorption mechanisms for biochar, CMBC, and P_s_-CMBC included electrostatic attraction and surface complexation; additionally, for P_s_-CMBC, Cd (II)-π interactions and coordination of Cd (II) with P=O were critical mechanisms for Cd (II) removal. The results of this study demonstrate that phosphate-loaded CMBC can be used as an effective treatment for heavy metal pollution in aqueous media.

## 1. Introduction

In recent years, contamination with heavy metals, such as cadmium (Cd), arsenic (As), lead (Pb), and stibium (Sb), has attracted worldwide concern, due to their high toxicity and non-biodegradability [1,2]. Cd, one of the most toxic heavy metals, is discharged into the environment via paints, waste batteries, mining, and other industrial processes; moreover, Cd can easily migrate and enrich agricultural crops [3,4]. Furthermore, Cd can cause serious health problems in wildlife and humans via the food chain. For example, long-term exposure to Cd can result in damage to both the bones and kidneys [5,6]. The US Environmental Protection Agency (EPA) has established Cd limit standards for drinking water as being less than 5 μg·L^−1^ [7]. Thus, it is imperative to develop an appropriate technique for efficient and selective Cd (II) removal from wastewater.

Chemical methods such as membrane filtration, adsorption, and ion exchange are applied to control Cd (II) pollution in aqueous media. Among them, adsorption has gained interest, owing to its simple operation, low cost, high efficiency, and economic feasibility for practical applications [8,9]. In recent years, carbon-rich sorbent materials, including activated carbon, carbon fibers, graphene nanomaterials, and other carbon-based materials, have gained attention [10,11]. However, the high cost of these advanced materials may hinder their practical application in heavy metal remediation. Owing to the developed micropore structure, extensive specific surface area, and multiple functional groups, biochar has received increasing attention as an eco-friendly, cost-effective, and carbon-based material [12]. It has been demonstrated that the synthesis of biochar is conducive to carbon sequestration, waste management, environmental remediation, and improvement in soil properties, owing to its high pH and rich matter content [13,14]. Heavy metal ions could be adsorbed onto biochar via electrostatic attraction due to negative charges on the surface of biochar [15]. In addition, the raw materials used to prepare biochar may contain traces of heavy metals. So, the environmental risks of biochar synthesized via pyrolysis also require attention [16]. Xu [17] found that the heavy metal risk of kitchen waste biochar decreased as pyrolysis temperature increased from 300 to 600 °C, and the biochar pyrolyzed at 500 °C was the most suitable for the eco-friendly, efficient and cost-saving remediation of Cd (II)-polluted water. A previous study has shown that the contaminant adsorption ability of pristine biochar is limited because of its poor selectivity and non-ideal physicochemical property [5]. Thus, biochar modification using other reagents is needed to further improve its Cd (II) adsorption performance. Several researchers have prepared new functional absorbent materials by adding chemical reagents such as HCl, magnesium oxide, sulfur, iron, and manganese to modify raw biochar [1,12,18]. In addition, a two-step process of pyrolysis and activation with different chemical reagents is usually applied to achieve a more efficient adsorption capacity for heavy metal contaminants [19,20]. Previous studies have confirmed that phosphate-containing materials exhibit excellent performance in heavy metal removal from solutions using phosphate-modified biochar [21,22]. Peng [5] synthesized phosphorus-modified biochar using phosphoric acid-modified pine wood chip biochar and achieved a larger surface area, a higher amount of oxygen-containing functional groups, and better sorption capacity of Cu (II) and Cd (II) compared to virgin biochar. Phosphorus-modified biochar prepared with rape straw as the raw material and orthophosphate as the modifying reagent showed outstanding performance for Pb sorption in wastewater [22]. Zhang [16] applied a P-modified biochar to heavy-metal-contaminated soil and found that higher ash and P retention on the P-modified biochar endorsed the conversion of Cu and Cd into more stable forms, and greatly reduced the extraction of Cd and Cu from soil. In addition, uranium (VI) removal from a solution using phosphate-functionalized bamboo biochar has also been reported [23]. Phosphorus-modified biochar has outstanding removal performance for heavy metals, which may be due to the phosphate combining with heavy metal ions to form mineral precipitates, while the heavy metal ions could interact with the active functional groups to generate more stable metalorganics [24]. Studies focused on Cd (II) sorption via phosphate-loaded Ca-Mn-impregnated biochar (P_s_-CMBC; prepared via the phosphate sorption process on CMBC) are limited. Our previous studies prepared Ca-Mn-impregnated biochar by modifying biochar with CaCl_2_ and KMnO_4_, which increased the adsorption active sites and microporous structure of CMBC and showed that CMBC had excellent sorption performance in phosphate removal from solution, and that CMBC possesses stable properties and strong buffering capacity [25]. Thus, we hypothesized that P_s_-CMBC may have more capacity for adsorption of Cd due to the presence of P in the biochar.

In this study, P_s_-CMBC was prepared via a phosphate adsorption process on Ca-Mn-impregnated biochar (CMBC). Moreover, a series of Cd adsorption experiments and characterization analyses of P_s_-CMBC were performed. The aims of this study were to (1) explore the basic properties of P_s_-CMBC, (2) evaluate the Cd (II) sorption behavior of P_s_-CMBC, and (3) determine the possible sorption mechanisms of Cd (II) on P_s_-CMBC via characterization.

## 2. Results and Discussion

### 2.1. General Properties of P_s_-CMBC

The results of space pore structure parameters, including the specific surface area, pore volume, and average pore diameter of P_s_-CMBC, are listed in Table 1. The SSAs of sorbents for P_25_-CMBC, P_50_-CMBC, and P_75_-CMBC were 81.63, 75.15, and 66.59 m^2^·g^−1^, respectively. The average pore diameter and pore volume decreased with the increase in P loading content (Table 1), which may be due to partial blockage in the biochar or the collapse of the pore structure due to P-Ca precipitation and complexation reactions during the process of phosphate sorption on CMBC.

Table 1 summarizes that all the P_s_-CMBC were alkaline, with the highest pH of 9.27 (P_50_-CMBC) and the lowest pH of 9.03 (P_25_-CMBC). This might be due to carbonates, the increase in ash contents generated, and the accumulation of alkaline inorganic matter at higher pyrolysis temperatures [26]. In addition, the presence of carboxyl and hydroperoxide functional groups on the surface of Ps-CMBC decreased with increasing temperature during the pyrolysis process [27,28]. Generally, the high aromatic structure of biochar may form more stable heavy metal complexing bonds, which could improve the sorption performance and reduce the risk of heavy metals [29]. The H/C ratio value may be applied to assess the degree of carbonization, which can indicate the degree of aromatization of biochar [30]. The H/C values of P_25_-CMBC, P_50_-CMBC, and P_75_-CMBC were 0.049, 0.050, and 0.051, respectively, indicating that orthophosphate improved the aromaticity of biochar [22].

### 2.2. Kinetics Experiment

**Figure 1:**
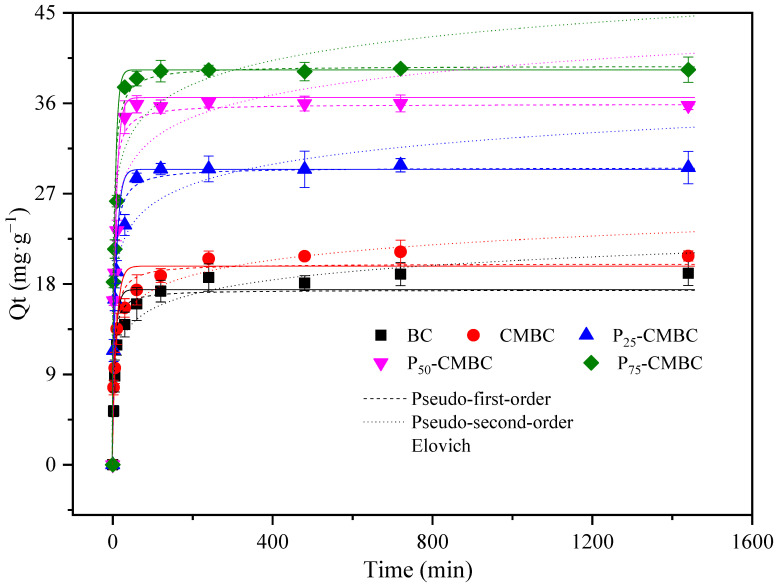
Adsorption kinetics of Cd on different adsorbents.

The time required for reaching adsorption equilibrium is an important factor in assessing sorbent efficiency. The adsorption capacity of Cd (II) on biochar with increasing contact time is listed in Figure 1. The Cd (II) sorption on P_s_-CMBC reached equilibrium within 2 h, which was less than the time required by biochar and CMBC (approximately 8 h). This result suggests that phosphate loading may accelerate Cd sorption. So, 24 h was chosen as the sorption time in the subsequent batch experiments to ensure that adsorption equilibrium was achieved. The Cd adsorption rate of biochar increased rapidly in the first 1–2 h, which is called the rapid initial phase. In the second (slow) phase, the adsorption rate declined from 2 h to 6 h, as the mass of the sorption sites was occupied [12].

To further evaluate the Cd (II) removal efficiency of different sorbents, three models (Supplementary Materials, Equations (S1)–(S3) have been used for fitting the kinetics curve [31].

Table 2 presents the corresponding parameters of the kinetic fitting models. In general, the theoretical adsorption amounts were in good agreement with the equilibrium experimental results. The pseudo-second-order model better fitted to kinetics data with respect to high correlation coefficients, implying that the reaction speed of Cd (II) removal by biochar is mainly dominated by chemisorption actions such as ion exchange and precipitation [12]. Moreover, the pseudo-second-order model also defines the processes of outer liquid film diffusion, surface sorption, and intra-particle diffusion actions [32].

### 2.3. Isotherm Experiments

**Figure 2:**
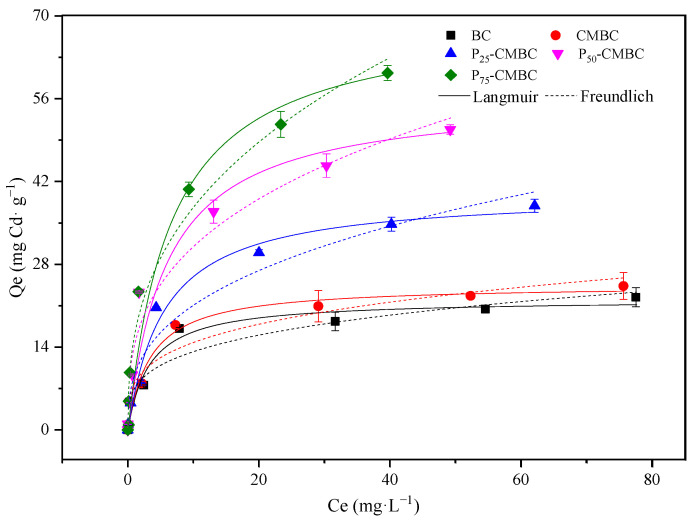
Adsorption isotherms of Cd onto various adsorbents.

As shown in Figure 2, the adsorption capacity of Cd (II) on biochar, especially for P_s_-CMBC, increased rapidly when increasing the initial content of Cd (II) in the solution. The Cd equilibrium adsorption capacity on various sorbents can be expressed as follows: biochar < CMBC < P_25_-CMBC < P_50_-CMBC < P_75_-CMBC.

To further elucidate the mechanism and sorption performance, Langmuir and Freundlich models were used, and the corresponding models can be seen in Supplementary Materials, Equations (S4)–(S6).

According to the fitting parameters, the Langmuir model fitted better than the Freundlich model (Table 3), indicating that Cd (II) sorption on biochar may be a monolayer coverage [31]. The corresponding values of maximum adsorption capacity fitted by the Langmuir model (Q_m_) of biochar, CMBC, P_25_-CMBC, P_50_-CMBC, and P_75_-CMBC are 22.05, 24.51, 40.01, 56.70, and 70.13 mg·g^−1^, respectively. It is clear that P_s_-CMBC exhibited a much higher adsorption capacity than the raw biochar, and the Q_m_ of P_25_-CMBC, P_50_-CMBC, and P_75_-CMBC increased 1.82, 2.57, and 3.18 times, respectively, compared to biochar. These results suggest that the phosphate-sorbed method can greatly enhance the Cd (II) sorption capacity of biochar. A previous study showed that the P-loaded method has good prospects in the commercial field for improving Cd(II) adsorption performance [33].

The maximum Cd sorption capacity of P_75_-CMBC (Q_m_: 70.1 mg·g^−1^) fitted via Langmuir was higher than other modified biochars like commercial active carbon (12.6 mg·g^−1^) [34] and synthetic hydroxyapatite nanoparticles (61.7 mg·g^−1^) [35]. In addition, Q_m_ is close to the biochars after HCl treatment (68.2 mg·g^−1^) [12] while smaller than hydroxyapatite-modified sludge-based biochar (114.7 mg·g^−1^) [36]. These results show that P-loaded Ca-Mn-impregnated biochar has a great potential as a sorbent for Cd(II) removal.

### 2.4. Zeta Potential of Biochar

**Figure 3:**
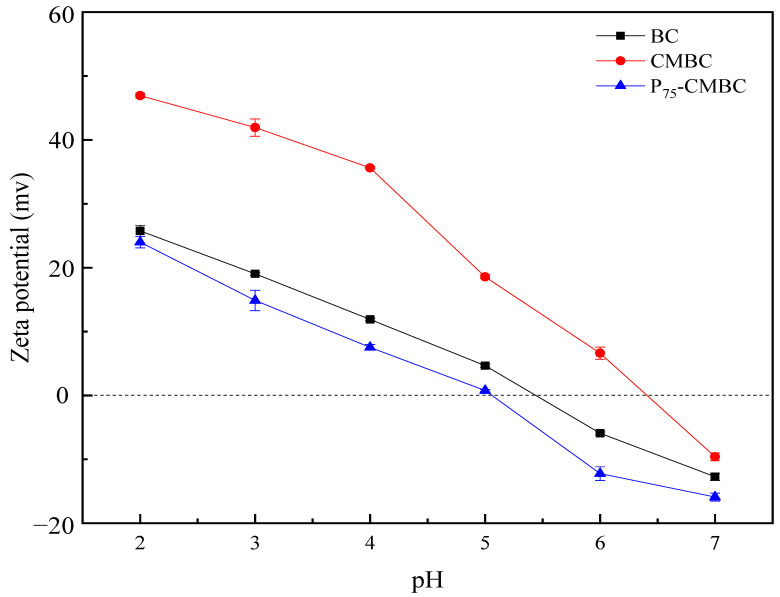
Zeta potential of BC, CMBC, and P_75_-CMBC.

The zeta potential is a crucial index of the dispersion stability of colloids. The sign (positive and negative) and the absolute value of the zeta potential indicate the level of electrostatic attraction or repulsion between charged particles in the dispersion [37]. Figure 3 shows that the zeta potential of different sorbents declined with increasing pH value, which may result from the various degrees of protonation of carboxyl and hydroxyl groups under different pH values. The oxygen-containing functional groups could be deprotonated under alkaline conditions, leading to a decrease in the zeta potential. The pHzpc value of P75-CMBC (pH 5.08) was lower than that of biochar (pH 5.53) and CMBC (pH 6.41). These results may be due to phosphate loading on the surface of the biochar, which increased the negative charge and reduced the zeta potential of the biochar’s surface effectively. Therefore, phosphate loading favors the adsorption of heavy metals from solutions [38].

### 2.5. Effect of pH

The initial pH value is a crucial factor in Cd adsorption performance because it can change the surface charge of the sorbent, the level of ionization, and the sorbate speciation [39]. As presented in Figure S1, the sorption performance of Cd on adsorbents is strongly related to the pH of the initial solution (3–7). As the pH of the solution changed from 3 to 7, the sorption amount of Cd was greatly enhanced, and a relatively constant Cd adsorption capacity was maintained at pH > 5.0. These results are consistent with those of previous studies [23,40]. In general, as the pH value of the solution increases, the deprotonation of biochar results in an increase in negative charges, which is conducive to enhancing the adsorption capacity of heavy metals via electrostatic action or precipitation. At a lower pH value (3) of the solution, the surface of the biochar was protonated and the positive charge on the surface of the biochar increased, which tended to repel the adsorption of positively charged Cd (II) ions for electrostatic repulsion [7]. Additionally, redundant protonation of the sorbent surface promotes competition between numerous H^+^ binding sites with heavy metal ions at low pH levels [41]. Under acidic conditions, we can predict that cadmium will detach from the material’s surface after initially binding to it, allowing for the material to be effectively recycled.

### 2.6. Adsorption Thermodynamics

To further assess the stability and feasibility of Cd adsorption on the adsorbents, the effects of temperature on Cd sorption were explored at 288.15, 298.15, and 308.15 K. As shown in Figure S2, the Cd sorption amounts improved with increasing reaction temperature (288.15, 298.15, and 308.15 K). This implies that higher temperatures have a positive effect on the Cd sorption capacity.

Three thermodynamic parameters, such as the standard entropy change (ΔS^0^), standard enthalpy change (ΔH^0^), and Gibbs free energy (ΔG^0^), were calculated using Supplementary Materials, Equations (S7)–(S9), respectively.

The parameter ΔG^0^ could estimate the thermodynamic potential and the level of spontaneity of the sorption. When the ΔH^0^ value is positive, the sorption process is endothermic, whereas it is negative for an exothermic process. Figure S2f shows a linear plot of 1/T versus ln (K_L_), and all the corresponding thermodynamic parameters are listed in Table 4. The negative values of ΔG^0^ suggest that the Cd sorption process is spontaneous. For instance, P_75_-CMBC showed a drop in ΔG^0^ value from −22.72 kJ·mol^−1^ to −25.79 kJ·mol^−1^ with increasing temperature from 15 to 35 °C. These results show that the spontaneity increases with increasing temperatures. The values of ΔH^0^ (8.07–18.66 kJ·mol^−1^) were positive for Cd sorption, indicating that the sorption process on the sorbents was endothermic, and the biochar would possess higher sorption capacity with the increasing temperature. Additionally, the positive values of ΔS^0^ (0.108, 0.124, and 0.143 kJ·mol^−1^·K^−1^ for the sorption on biochar, CMBC, and P_75_-CMBC, respectively) imply the spontaneity of Cd sorption. This result demonstrates that there was a great disorder of the adsorbate/adsorbent system when Cd was migrated from the aqueous phase to the biochar’s surface [42].

### 2.7. The Surface Characterization

The SEM images show the surface morphology of P_75_-CMBC (after Cd sorption) at different resolutions. Figure S3 shows an abundant pore structure. In addition, the SEM images also show the phenomenon of pore obstruction, which could be due to the crumble of the internal pore structure of the biochar. SEM-EDS investigation after Cd sorption confirmed phosphate loading by the intense P peaks present in P_75_-CMBC (wt.%: 4.74); Cd was also observed by EDS, demonstrating that Cd was successfully adsorbed on P-sorbed-biochar (wt%: 7.46). Other constituent elements, including C, O, Mn, and Ca, are shown in Figure S3.

**Figure 4:**
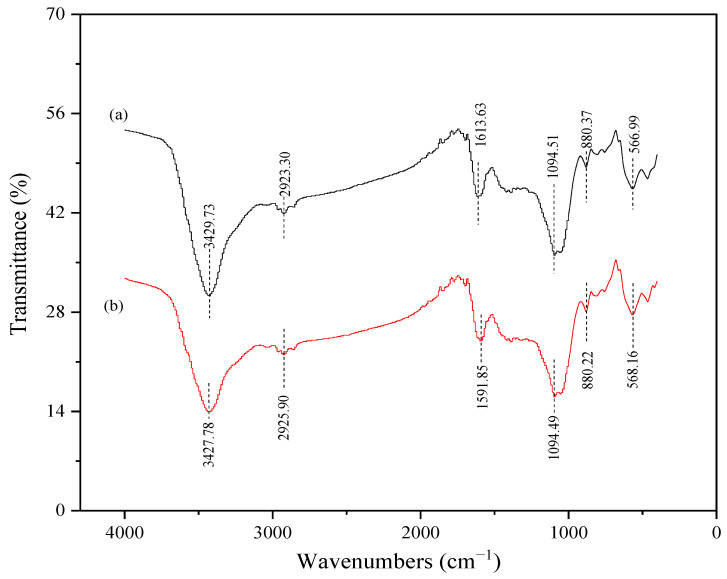
Fourier transform infrared spectrum (FTIR) of P_75_-CMBC before (a) and after (b) Cd (II) sorption.

The biochar surface’s functional groups had a crucial effect on its adsorption performance. The FTIR spectroscopy results of P_75_-CMBC before and after Cd adsorption are listed in Figure 4. The broad and strong peaks of the biochar observed at 3427–3430 cm^−1^ were related to hydroxyl band (-OH) [43]. The peaks observed at 2923–2926 cm^−1^ corresponded to the stretching of -CH_2_ vibrations, which is a characteristic peak of aromatic or aliphatic compounds [33]. The peaks observed at 1591–1614 cm^−1^ could be attributed to the stretching vibration of the C=C or C=O group of carboxyl [44]. A previous study showed that the aromatic structure serves as a π-donor during heavy metal adsorption. Functional groups such as C=C are important for Cd (II) sorption because they can interact with Cd (II) ions via Cd (II)-π interactions [5]. The bands located at approximately 1094.5 cm^−1^ may be ascribed to the vibration of C-O-C, or the ionization of P^+^-O^−^ in phosphate esters, and the vibration of P-O-P chains [45]. These results suggest that the P (P=O or P=OOH) loaded on the biochar may form compounds with heavy metal ions. This plays a crucial role in upgrading the sorption capacity of heavy metals. The weak peaks at approximately 802 cm^−1^ may be attributed to the stretching vibrations of C-H. Moreover, the peaks under 600 cm^−1^ could be attributed to the stretching vibrations of the metal halogen.

**Figure 5:**
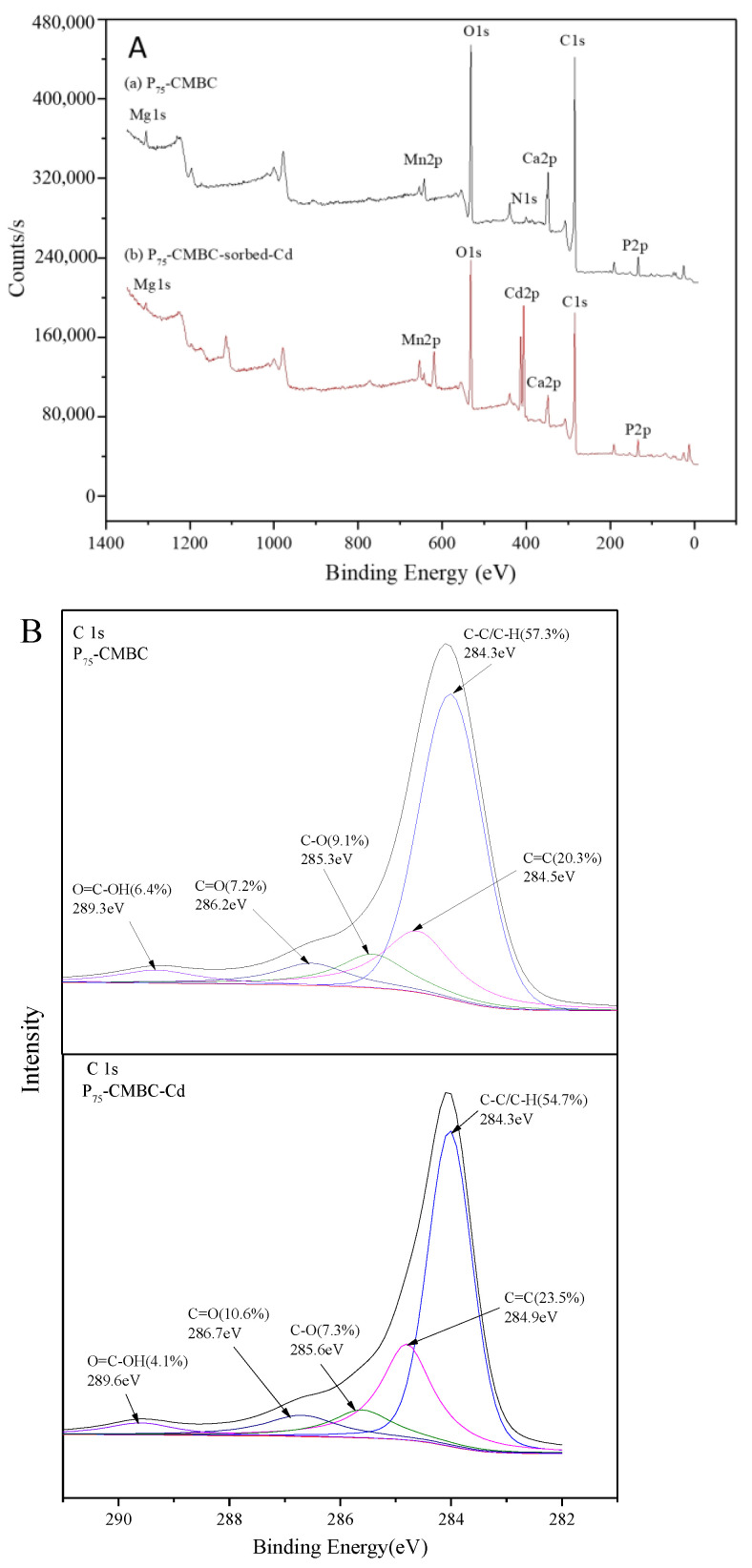

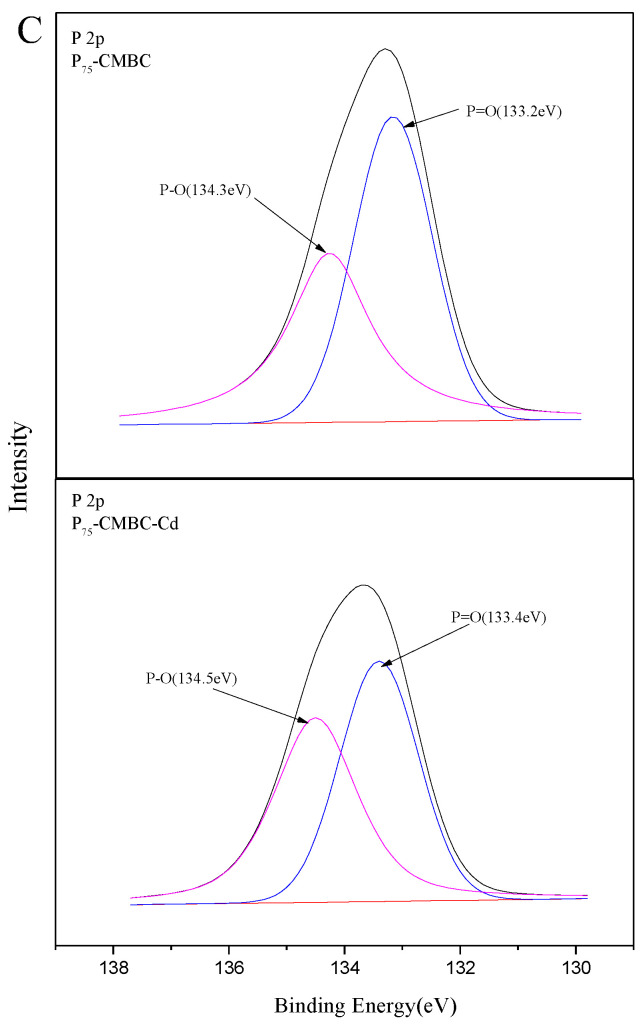
The XPS spectra of P_75_-CMBC before or after Cd (II) sorption: (**A**) XPS spectra of P_75_-CMBC before or after Cd (II) sorption. (**B**) C 1s XPS spectra of P_75_-CMBC before and after Cd (II) adsorption. (**C**) P 2p XPS spectra of P_75_-CMBC before and after phosphate adsorption.

Figure 5 shows the results of the XPS analysis. The peaks of C1s, O1s, Ca2p, N1s, Mn2p, Mg1s, and P2p can be observed in all spectra of P-loaded biochar, implying that P was successfully loaded onto the modified biochar (Figure 5A). In addition, a new peak at approximately 413.6 eV after Cd (II) sorption was observed, and the surface atomic Cd content of P_75_-CMBC was 1.82%. This suggests that Cd (II) was successfully adsorbed onto the surface of P_s_-CMBC. The XPS spectrum of C1s before and after Cd adsorption is shown in Figure 5B. Before Cd sorption, five peaks were observed at the following bond energies: C-C/C-H (284.3 eV, 57.3%), C=C (284.9 eV, 20.3%), C-OH/C-N (285.6 eV, 9.1%), C=O (286.7 eV, 7.2%), and O=C-OH (289.6 eV, 6.4%) [17]. After Cd sorption, the corresponding functional groups occupied 54.7%, 23.5%, 7.3%, 10.6%, and 4.1%, respectively. The decrease in O=C-OH and C-OH suggests that the process of Cd (II) sorption involves the depletion of carboxyl and hydroxyl groups on the surface of biochar. In addition, augmentation of the C=O functional groups indicated that the O atom may act as an electron donor during the uptake of Cd (II) [12]. For the P 2p spectrum of P-loaded biochar, the pristine peak could be fitted well by two peaks at about 133.2 eV and 134.3 eV, before and after Cd adsorption, respectively (Figure 5C). This indicates that P may combine with O atoms to form P-O and P=O groups during the phosphate adsorption process [23]. Additionally, the corresponding groups were considerably weakened after Cd adsorption, implying that chemical reactions between Cd (II) and P compounds may have occurred on the surface of P_75_-CMBC [16].

Thus, the sorption mechanisms of Cd (II) on P_s_-CMBC involve the following: (1) Electrostatic attraction occurs between the positively charged Cd (II) in solution and negatively charged surface of P_s_-CMBC. (2) The P (P=O or P=OOH) loaded on the biochar forms complex compounds with Cd (II). (3) The electronic structure of P-loaded biochar provides a cation–π bond between Cd (II) and a π-electron donor. (4) The coordination of Cd (II) with O atoms of P=O/P=OOH occurs via filling the empty orbital of Cd (II) with P=O groups.

## 3. Materials and Methods

### 3.1. Reagents and Analysis Equipment

All chemical reagents used in this study, including calcium chloride (CaCl_2_), potassium permanganate (KMnO_4_), sodium dihydrogen phosphate (NaH_2_PO_4_), cadmium nitrate tetrahydrate (Cd (NO_3_)_2_·4H_2_O), sodium nitrate (NaNO_3_), hydrochloric acid (HCl), and sodium hydroxide (NaOH), were of analytical grade; all solutions were prepared and biochar washing was carried out with deionized (DI) water. The Cd (II) was determined using an atomic absorbance spectrometer via the flame method.

### 3.2. Ca-Mn-Impregnated Biochar (CMBC) Preparation

The CMBC was synthesized via a two-stage pyrolysis and impregnation process. Firstly, corn straw powder was pyrolyzed in a muffle furnace for 1 h at 600 °C under a sufficient N_2_ environment. The obtained biochar was washed and dried to obtain pristine biochar. For biochar modification, a 40 mL solution containing 20 mL CaCl_2_ (25.53 g·L^−1^) and 20 mL KMnO_4_ (7900 mg·L^−1^) was prepared for impregnation of 5 g of biochar, and the mixture was stirred on a magnetic stirrer followed by water bath drying. Then, the CMBC was obtained by pyrolyzing the dried mixture at 600 °C. Finally, P_s_-CMBC was prepared via the phosphate adsorption process on CMBC. Phosphate sorption experiments were conducted by adding 2 g of CMBC to 1 L beakers containing 500 mL of P solutions (the sorbent dosage was 4 g·L^−1^; initial phosphate concentration was 25, 50, or 75 mg·L^−1^). The mixture stirring was performed at 25 ± 0.5 °C and 300 rmp. The mixture was then filtered and the P_s_-CMBC was dried to a constant weight at 60 °C in an oven. The P_s_-CMBCs prepared with initial phosphate concentrations of 25, 50, and 75 mg P·L^−1^ can be denoted as P_25_-CMBC, P_50_-CMBC, and P_75_-CMBC, respectively.

### 3.3. Characterization

An elemental analyzer (Elementar VARIOEL cube, Langenselbold, Germany) was used to measure the total elemental compositions, such as those of S, N, O, H, and C. The pH of biochar was determined with a pH meter (Orion Star A215, Thermo Scientific, Waltham, MA, USA). The Brunauer–Emmett–Teller (BET) method was used to determine specific surface area (SSA), pore volume, and pore diameter distribution. The zeta potential of prepared materials was investigated using a Malvern Zetasizer NanoZSE (Nano ZS90, Malvern, UK).

The elemental composition and surface morphology of the biochar were analyzed using a scanning electron microscope coupled with an energy-dispersive spectrometer (SEM-EDS) analyzer (ZEISS, Gemini SEM 500, Oberkochen, Germany). The surface elemental content (C, O, Ca, Mn, P, and Cd) and the binding energy on the biochar surface were characterized via X-ray photoelectron spectroscopy (XPS; PHI 5000 Versa Probe, Chigasaki, Japan). The functional groups on the surface of biochar were characterized via Fourier-transform infrared spectroscopy (FTIR; Thermo Scientific Nicolet iS5, Waltham, MA, USA).

### 3.4. Adsorption Experiments

The Cd stock solution (1000 mg·L^−1^) was prepared using Cd(NO_3_)_2_·4H_2_O, and 85 mg·L^−1^ NaNO_3_ was used as the background electrolyte. The solution of the required initial concentration was obtained by diluting the stock solution. Batch sorption studies were conducted to investigate the effects of temperature, solution pH, initial Cd^2+^ concentration, and contact time on the Cd^2+^ sorption efficiency and performance. The solution pH values were adjusted using 4000 mg·L^−1^ NaOH or 6300 mg·L^−1^ HNO_3_ solutions. All sorption experiments were conducted three times, and standard deviation error bars were obtained from three replicates.

#### 3.4.1. Kinetics Experiment

The sorption kinetics of Cd^2+^ were studied by adding 0.25 g of biochar to a 500 mL beaker containing 250 mL Cd^2+^ solution (50 mg·L^−1^) followed by stirring at 300 rpm, and the temperature was set at 25 ± 0.5 °C for different sampling times (0, 2, 5, 10, 30, 1, 2, 4, 8, 12, and 24 h). A 0.5 mL solution was filtered and the remaining Cd^2+^ concentration in the filtrate was measured using an atomic absorption spectrometer (AAS; Beijing Haiguang Instruments, Co., Ltd., Beijing, China), using the flame method, where the sensitivity was 0.05 mg·L^−1^ and a minimum detectable limit of 0.05 mg·L^−1^ was used for Cd. The Cd sorption amounts at time t were calculated using Equation (1):

(1) $$Q_{t}=\frac{\left(C_{o}-C_{t}\right)\cdot V}{m}$$

where the Cd sorption amount at time t is represented by Q_t_ (mg·g ^−1^), C_o_ (mg·L^−1^) and C_t_ (mg·L^−1^) are the initial Cd^2+^ concentrations at time t (min), respectively, V (mL) represents the volume of Cd solution, and the mass of adsorbents is represented by m (g).

#### 3.4.2. Isotherms Experiment

Adsorbents of 1 g·L^−1^ (0.02 g of biochar in 20 mL of Cd solution) were taken in 40 mL brown glass vials containing 20 mL of different initial Cd concentrations (0, 1, 5, 10, 25, 50, 75, and 100 mg Cd·L^−1^). The vials were shaken (end-to-end) for 24 h at a temperature of 25 ± 0.5 °C at 120 rpm. At sorption equilibrium, the mixtures were completely filtered through a 0.22 μm membrane and the remaining Cd^2+^ concentration was measured using an AAS. The Cd sorption capacity at equilibrium Q_e_ (mg·g^−1^) was calculated using Equation (2):

(2) $$Q_{e}=\frac{\left(C_{o}-C_{e}\right)\cdot V}{m}$$

where the Cd sorption capacity at equilibrium is Q_e_ (mg·g^−1^), the equilibrium and initial concentrations of Cd are represented by C_e_ (mg·L^−1^) and C_o_ (mg·L^−1^), respectively, m (g) is the mass of the adsorbent and V (mL) is the volume of the solution.

#### 3.4.3. Effect of pH on Cd Adsorption

The influence of solution pH was determined with various initial pH values (3, 4, 5, 6, and 7) of 20 mL Cd solution (0, 1, 5, 10, 25, 50, 75, and 100 mg Cd·L^−1^). Adsorbents (0.02 g) were added in the solution of Cd (1 g·L^−1^) and shaken for 24 h at 120 rpm (end-to-end).

#### 3.4.4. Adsorption Thermodynamics

For thermodynamic analyses, adsorption was carried out at temperatures of 15, 25, and 35 °C. The sorption process was followed by an isothermal sorption method: 0.1 g of prepared materials was added into 40 mL brown glass vials that contained 20 mL of various initial Cd concentrations (0, 1, 5, 10, 25, 50, 75, and 100 mg Cd·L^−1^). The equilibrium Cd concentrations were then determined.

### 3.5. Data Analysis

All experimental data are the average of triplicate measurements. Statistical analysis was performed using Microsoft Excel 2010, and the results are presented as mean ± standard deviation and were plotted using Origin 2016 software for Windows (Origin Lab, Northampton, MA, USA).

## 4. Conclusions

In this study, P-loaded biochar was prepared to elucidate the sorption mechanisms of Cd from aqueous solutions. P_s_-CMBC showed higher sorption efficiency for a shorter time, compared to biochar and CMBC, and exhibited outstanding Cd (II) adsorption performance, with an equilibrium sorption capacity of 70.13 mg·g^−1^, higher than those of biochar (22.05 mg·g^−1^) and CMBC (24.51 mg·g^−1^). Higher pH (5–7) had relatively lower effects on the Cd (II) sorption capacity. The primary mechanisms for Cd adsorption on P_s_-CMBC were surface complexation, electrostatic attraction, Cd (II)-π interactions, and the coordination of Cd (II) with P=O. In summary, P-sorbed biochar has great potential for Cd (II) adsorption.

## Figures and Tables

**Table 1:** The basic physiochemical properties of raw biochar Ca/Mn-impregnated biochar, and P-sorbed biochar.

Adsorbent	Physical Properties				Elemental Contents (%)						H/C
	S_BET_ (m^2^·g^−1^)	Pore Volume (cm^3^·g^−1^)	Pore Size (nm)	pH	C	O	H	N	S	Ash	
BC	43.42	0.041	1.569	9.88	71.60	20.96	3.33	2.93	0.18	15.03	0.046
CMBC	162.01	0.131	2.558	9.62	68.25	24.95	2.97	2.66	0.17	21.57	0.044
P_25_-CMBC	81.63	0.079	1.806	9.03	67.32	26.61	3.27	1.62	0.31	17.82	0.049
P_50_-CMBC	75.15	0.061	1.695	9.27	65.94	27.99	3.32	1.85	0.25	18.26	0.050
P_75_-CMBC	66.59	0.063	1.688	9.15	64.14	29.67	3.29	1.61	0.59	17.53	0.051

**Table 2:** The kinetic parameters for Cd sorption on various adsorbents.

Adsorbents	Pseudo-First-Order			Pseudo-Second-Order			Elovich		
	Q_e_ (mg·g^−1^)	K_1_	R^2^	Q_e_ (mg·g^−1^)	K_2_	R^2^	*α*	*β*	R^2^
BC	17.43	0.137	0.946	17.39	0.018	0.952	41.66	0.489	0.951
CMBC	19.71	0.105	0.914	19.95	0.012	0.972	74.79	0.470	0.965
P_25_-CMBC	29.12	0.113	0.932	29.30	0.009	0.985	273.65	0.357	0.931
P_50_-CMBC	36.10	0.121	0.941	35.47	0.011	0.964	832.59	0.318	0.896
P_75_-CMBC	38.81	0.157	0.951	39.18	0.009	0.977	1149.4	0.298	0.908

**Table 3:** Langmuir and Freundlich equation parameters of Cd adsorption onto different adsorbents.

Adsorbents	Langmuir			Freundlich		
	Q_m_ (mg·g^−1^)	K_L_ (L·mg^−1^)	R^2^	1/n	K_F_	R^2^
BC	22.05	0.303	0.961	0.275	7.029	0.927
CMBC	24.51	0.292	0.973	0.273	7.896	0.947
P_25_-CMBC	40.01	0.184	0.983	0.354	9.336	0.954
P_50_-CMBC	56.70	0.168	0.955	0.281	17.403	0.957
P_75_-CMBC	70.13	0.193	0.971	0.306	20.042	0.958

**Table 4:** Thermodynamic parameters for Cd adsorption on different adsorbents.

Adsorbents	Temperature (K)	ΔG^0^ (kJ/mol)	ΔH^0^ (kJ/mol)	ΔS^0^ (kJ/(mol·K))
BC	288.15	−24.66	8.07	0.108
	298.15	−25.89		
	308.15	−26.93		
CMBC	288.15	−24.56	11.16	0.124
	298.15	−25.78		
	308.15	−27.04		
P_25_-CMBC	288.15	−23.33	12.03	0.123
	298.15	−24.63		
	308.15	−25.79		
P_50_-CMBC	288.15	−23.08	17.22	0.139
	298.15	−24.41		
	308.15	−25.88		
P_75_-CMBC	288.15	−22.72	18.66	0.143
	298.15	−24.16		
	308.15	−25.59		

## Data Availability

All data generated or analyzed during this study are included in this published article and its Supplementary Materials.

## Supplementary Materials

The following supporting information can be downloaded at https://www.mdpi.com/article/10.3390/molecules28227553/s1, Figure S1: Effects of initial pH (3–7) on Cd adsorption of different materials. (A) BC; (B) CMBC; (C) P_75_-CMBC; Figure S2: Cd sorption of different materials at different temperatures. BC (a), CMBC (b), P_25_-CMBC (c) and P_50_-CMBC (d), P_75_-CMBC (e). Plots of lnb versus 1/T for different adsorbents (f); Figure S3: SEM images and EDS spectra of P_75_-CMBC after adsorbed Cd(II);
