# Supplementary material for: Effective Removal of Cd from Aqueous Solutions Using P-Loaded Ca-Mn-Impregnated Biochar
Source: Molecules. 2023 Nov 12;28(22):7553. doi: 10.3390/molecules28227553 (PMC10673148; doi:10.3390/molecules28227553)
Supplement: Supplementary file 1 [file molecules-28-07553-s001.zip › molecules-2686570-supplementary.pdf]

Supporting Information

Effective Removal of Cd from Aqueous Solutions Using P-loaded Ca-Mn-impregnated Biochar

Cheng Qiu [1], Chengwei Wang [2,3], Qinghai Liu [1], Minling Gao [2,*] and Zhengguo Song [2,4,*]

[1] Institute of Agricultural Product Quality Standard and Testing Research, Tibet Academy of Agricultural and Animal Husbandry Sciences, Lhasa 850032, China; chengqiu_2006@163.com (C.Q.); song199611@tom.com (Q.L.)
[2] Department of Civil and Environmental Engineering, Shantou University, No. 243 Daxue Road, Shantou 515063, China; wangcw96@163.com
[3] School of Environmental Science and Engineering, Tiangong University, No. 399 Binshui West Road, Xiqing District, Tianjin 300387, China
[4] Guangdong Provincial Key Laboratory of Marine Disaster Prediction and Prevention, Shantou University, Shantou 515063, China
* Correspondence: mlgao@stu.edu.cn (M.G.); zgsong@stu.edu.cn (Z.S.)

2. Materials and methods

2.1 Models

To further evaluate the Cd (II) removal efficiency of different sorbents, following models have been used for fitting the kinetics curve, represented as follows:

Pseudo-first-order model:

$$\mathrm{Q_t} = \mathrm{Q_e}(1 - e^{-bx}) \quad (3)$$

Pseudo-second-order model:

$$\mathrm{Q_t} = \frac{K_2 \mathrm{Q_e^2} t}{1 + \mathrm{k_2 Q_e^2} t} \quad (4)$$

Elovich model:

$$Q_t = \frac{1}{\beta}\ln(1 + \alpha \times \beta \times t) \quad (5)$$

where *t* is the contact time, the sorption amount of Cd at time t is Q_t (mg·g^{-1}), k_2 (g·mg^{-1}·min^{-1}) and k_1 (min^{-1}) are the sorption rate constants of the pseudo-second order and pseudo-first order, respectively, the equilibrium sorption capacity is Q_e (mg·g^{-1}). β is the desorption constant (g·mg^{-1}), and α represents the initial sorption rate (mg·g^{-1}·h^{-1}).

the Langmuir, Freundlich, and Sips models were used, and the corresponding models can be expressed as Eqs. (6), (7), and (8).

$$Q_e = \frac{Q_m K_L C_e}{1 + K_L C_e} \quad (6)$$

$$R_L = \frac{1}{1 + K_L C_o} \quad (7)$$

$$Q_e = K_F Ce^{1/n} \quad (8)$$

where Q_m (mg·g^{-1}) is the theoretical maximum sorption capacity, Q_e (mg·g^{-1}) and C_e (mg·L^{-1}) are the adsorption amount of Cd^{2+} at equilibrium and the residual Cd concentration at equilibrium, respectively, K_L (L·mg^{-1}) and K_F (L·g^{-1}) represent the

Three thermodynamic parameters, such as the standard entropy change (ΔS^0), standard enthalpy change (ΔH^0), and Gibbs free energy (ΔG^0) were calculated using Eq. (9), (10), and (11), respectively.

$$\Delta G^0 = -RT\ln K_L \quad (9)$$

$$\Delta G^0 = \Delta H^0 - T\Delta S^0 \quad (10)$$

The values of ΔH^0 can be expressed by the Van't Hoff equation as follows:

$$\ln K_L = -\frac{\Delta H^0}{RT} + \frac{\Delta S^0}{R} \quad (11)$$

where K_L is equilibrium constant of adsorption, the absolute temperature (K) is T, and the universal gas constant is R (8.314 J·mol^{-1}·K^{-1}). The slope and intercept of the linear plot of ln (K_L) versus 1/T were used to determine the values of entropy (ΔS^0) and enthalpy (ΔH^0).

Figure S

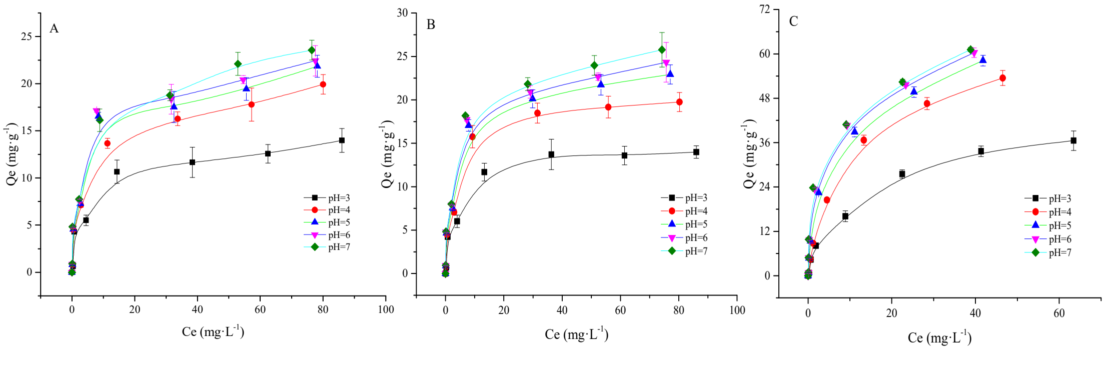

Fig.S1 Effects of initial pH (3 - 7) on Cd adsorption of different materials. (A) BC; (B) CMBC; (C) P_{75}-CMBC.

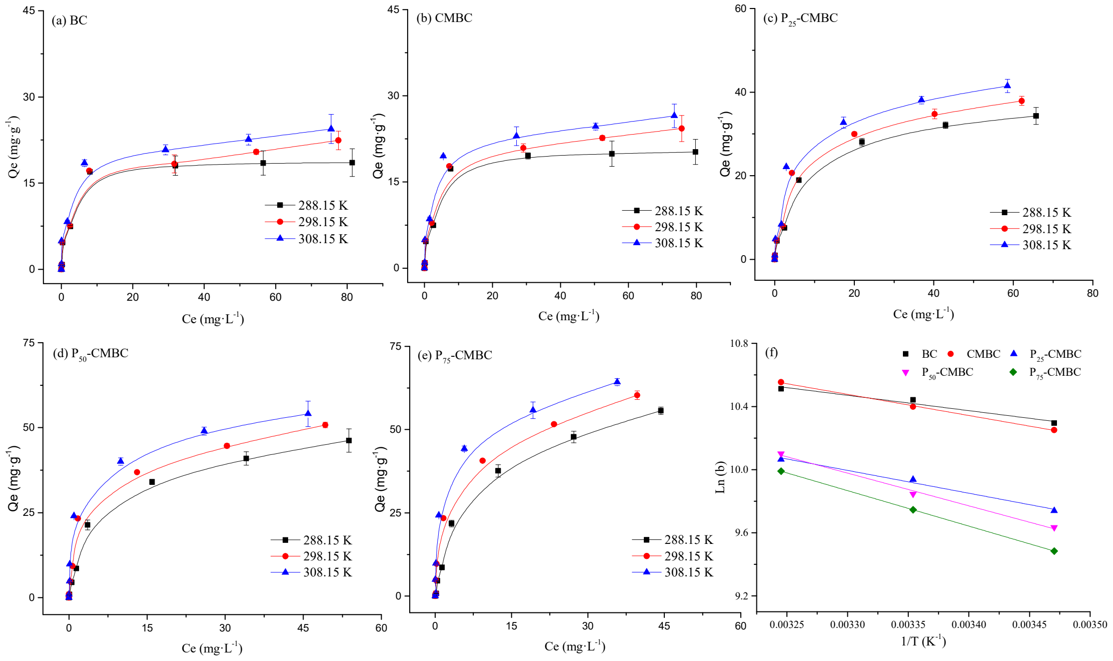

Fig. S2 Cd sorption of different materials at different temperatures. BC (a), CMBC (b), P_{25}-CMBC (c) and P_{50}-CMBC(d), P_{75}-CMBC (e). Plots of lnb versus 1/T for different adsorbents (f).

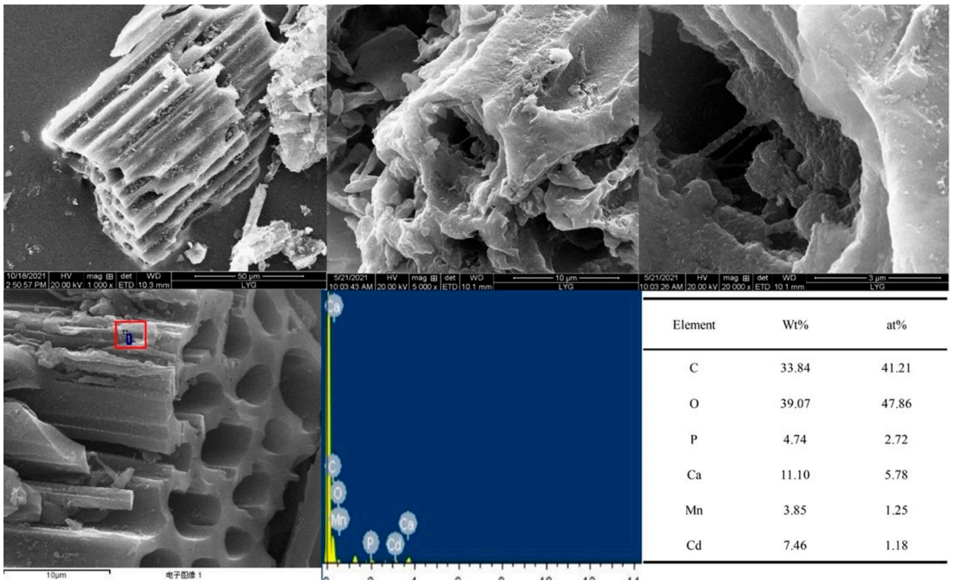

Element	Wt%	at%
C	33.84	41.21
O	39.07	47.86
P	4.74	2.72
Ca	11.10	5.78
Mn	3.85	1.25
Cd	7.46	1.18

Fig. S3 SEM images and EDS spectra of P_{75}-CMBC after adsorbed Cd(II)
